# Fibronectin glycation increases IGF-I induced proliferation of human aortic smooth muscle cells

**DOI:** 10.1186/1758-5996-4-19

**Published:** 2012-05-03

**Authors:** Maria Lúcia Corrêa-Giannella, Maria Regina Andrade de Azevedo, Derek LeRoith, Daniel Giannella-Neto

**Affiliations:** 1 Laboratory for Cellular and Molecular Endocrinology (LIM-25). Hospital das Clínicas da Faculdade de Medicina da Universidade de São Paulo, Av. Dr. Arnaldo, 455, Sala 4305, São Paulo, Brazil; 2 Universidade Santo Amaro (UNISA), R. Enéas de Siqueira Neto, 340, São Paulo, Brazil; 3 Division of Endocrinology and Diabetes, Department of Medicine, The Mount Sinai School of Medicine, 1 Gustave Levy Place, Box 1055, New York, USA; 4 Laboratory for Clinical and Experimental Gastroenterology (LIM-07). Hospital das Clínicas da Faculdade de Medicina da Universidade de São Paulo, Av. Dr. Arnaldo, 455, Sala #4387, São Paulo, Brazil

**Keywords:** Diabetes mellitus, Advanced glycation end products (AGE), Smooth muscle cells, PDGF, IGF-I, IGFBP-4

## Abstract

The advanced glycation end products, namely AGEs, contribute to long-termed complications of diabetes mellitus, including macroangiopathy, where smooth muscle cells (SMC) proliferation stimulated by platelet-derived growth factor (PDGF) isoforms and insulin-like growth factor-I (IGF-I) plays an important role. The objective of the present study was to investigate the effect of an AGE-modified extracellular matrix protein on IGF-I induced SMC proliferation and on the IGF-I-IGF binding protein 4 (IGFBP-4) axis under basal conditions and after stimulation with PDGF-BB. IGF-I resulted in significantly higher thymidine incorporation in SMC seeded on AGE-modified fibronectin (AGE-FN) in comparison to cells seeded on fibronectin (FN). This augmented proliferation could not be accounted for by increased expression of IGF-IR, by decreased secretion of IGFBP-4, a binding protein that inhibits IGF-I mitogenic effects or by increased IGF-IR autophosphorylation. PDGF-BB did not modulate IGF-IR and IGFBP-4 mRNA expression in any of the substrata, however, this growth factor elicited opposite effects on the IGFBP-4 content in the conditioned media, increasing it in cells plated on FN and diminishing it in cells plated on AGE-FN. These findings suggest that one mechanism by which AGE-modified proteins is involved in the pathogenesis of diabetes-associated atherosclerosis might be by increasing SMC susceptibility to IGF-I mitogenic effects.

## Background

Both type I and type II diabetes are powerful and independent risk factors for coronary artery disease, stroke, and peripheral arterial disease [1,2]. Prolonged exposure to hyperglycemia is recognized as the primary casual factor in the pathogenesis of diabetic complications [3,4]. Hyperglycemia induces a large number of alterations in vascular tissue that potentially promote accelerated atherosclerosis. Glycation of proteins is an important biochemical mechanism by which glucose mediates tissue damage, leading to the generation of advanced glycation endproducts (AGEs) and modifying the structure and function of several proteins, such as those which comprise extracellular matrixes [5]. It has been demonstrated that AGE formation alters some functional properties of collagen [6], vitronectin [7], laminin [8], and fibronectin (FN) [9], affecting their self-assembly and their binding to each other. AGEs can also induce synthesis and secretion of cytokines and growth factors after binding to AGE receptors (RAGE) in different cell types [7]. Monocytes exposed to AGE-modified matrix release tumor necrosis factor-α (TNF-α) [10], platelet-derived growth factor (PDGF) [11] and insulin-like growth factor-I (IGF-I) [12]. In vascular smooth muscle cells (SMC) AGE-RAGE interaction has been shown to activate cell signalling pathways linked to expression of inflammatory and prothrombotic genes, such as ERK1/ERK2 kinases and NF-kB [13].

The SMC, which constitute the medial layer of arteries, are normally in a differentiated contractile phenotype, but during the development of atherosclerotic lesions, a subpopulation of SMC is converted to a synthetic phenotype that is able to migrate and proliferate. Extracellular matrix proteins actively participate in this process, affecting SMC phenotype and modulating the cellular response to growth-regulatory molecules [14]. FN, which is found in increased amounts in early atherosclerotic plaques [15,16], can interact with cell surface receptors and promote the conversion of SMC to the synthetic state [17] and growth factors such as PDGF and IGF-I will act, respectively, as competence and progression factors for cell replication [18], exerting synergistic effects on SMC proliferation [19]. IGF-I is synthesized by vascular SMC where it is regulated by several factors, such as PDGF [20-22]. The final biological activity of IGF-I is determined by the number and affinity of its receptors (IGF-IR) as well as by its binding proteins (IGFBP) [23]. IGFBP-4, whose secretion is also modulated by PDGF [22,24], is one of the predominant IGFBPs produced by vascular SMC in culture, where it has an inhibitory effect on IGF-I- induced DNA synthesis [25].

The present study examined the effect of AGE-modified FN on IGF-I induced SMC proliferation as well as on the expression of IGF-IR and IGFBP-4 and their modulation by PDGF-BB, in order to investigate pathways that could be involved in diabetic macroangiopathy.

## Materials and methods

This study was approved by the Institution’s Ethic Committee (Comitê de Ética em Pesquisa do Hospital das Clínicas da Faculdade de Medicina da Universidade de São Paulo).

### Materials

Fibronectin and other routine reagents were obtained from Sigma Chemical Co. (St. Louis, MO), IGF-I and Des (1–3) IGF-I were supplied by GroPep (Adelaide, South Australia), PDGF-BB and all materials for cell culture were obtained from Life Technologies (Gaithersburg, MD). Iodinated [^125^I] des (1–3) IGF-I, [^125^I] labelled Protein A (100 mCi/mL) and ECL detection kit were purchased from Amersham (Arlington Heights, IL). Antibody 1.2, a monoclonal antibody to the C-terminus of the IGF-I receptor, was a kind gift from Dr. Kenneth Siddle (Cambridge, UK). Anti-IGFBP-4 antibody was obtained from Upstate Biotechnology, Inc. (Lake Placid, NY). Anti-phosphotyrosine antibody (RC20H) conjugated to horseradish peroxidase was purchased from BD Transduction Laboratories (Lexington, KY).

### Preparation of AGE-modified FN

**Figure 1:**
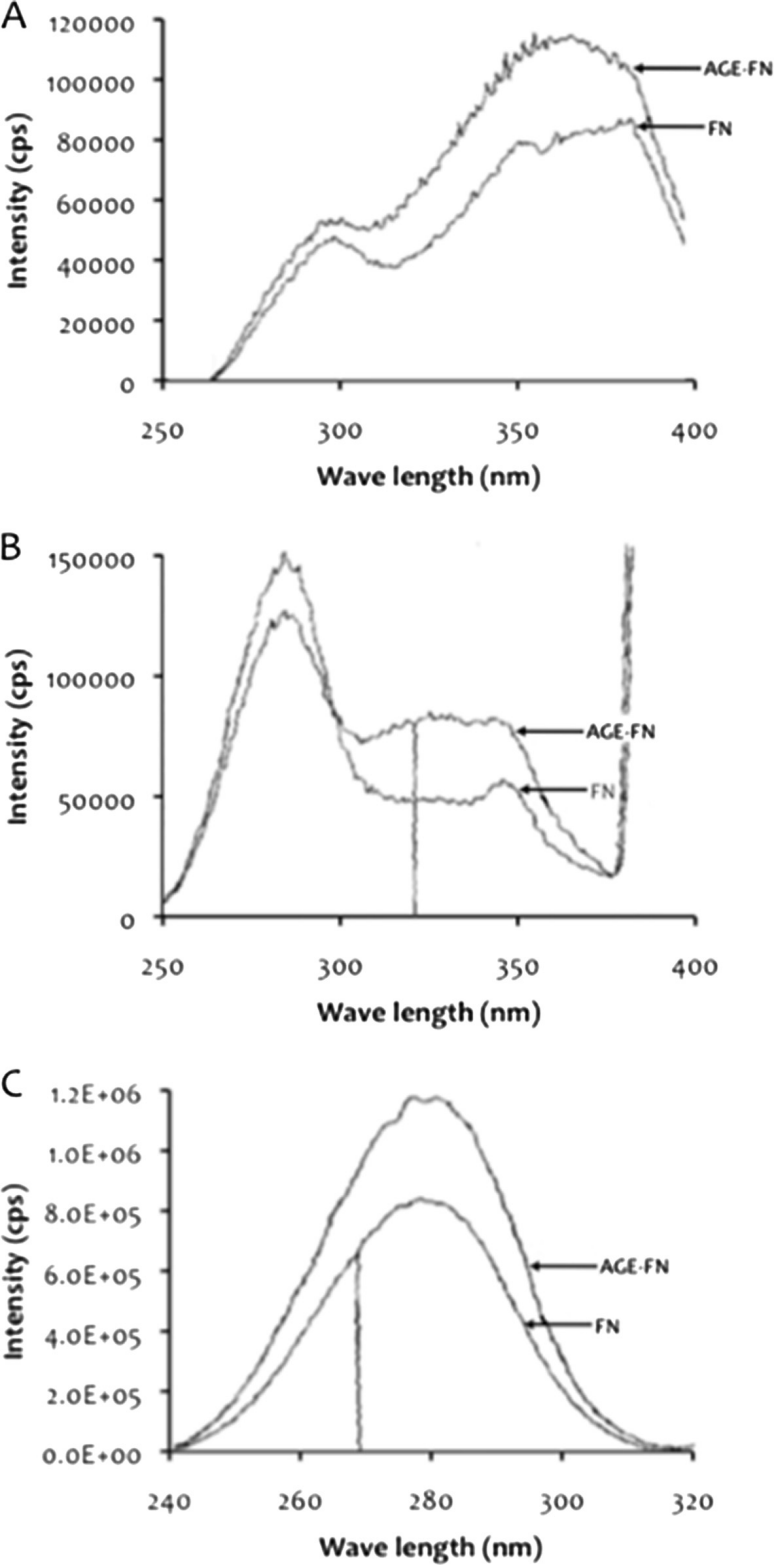
**Characterization of AGE-modified FN (AGE-FN).** Fluorescence of the AGE compound FFI observed at 440 nm after excitation at 375 nm **(Panel A)**. Fibronectin (FN) structural modification secondary to advanced glycation was established by assessing bityrosine formation (400 nm upon excitation at 325 nm) **(Panel B)** and tryptophan quenching (334 nm upon excitation at 275 nm) **(Panel C)**.

FN (2.5 mg) was incubated in phosphate-buffered saline (PBS) (pH 7.4) with 500 mM D-glucose at 37°C in the presence of protease inhibitors (1.5 mM phenylmethylsulfonyl fluoride [PMSF], 0.5 mM EDTA) and antibiotics (100 U/mL penicillin, 100 μg/mL streptomycin) for 6 weeks. Control FN was incubated under the same conditions without glucose. Unreacted sugar was removed by dialysis against PBS. Characteristic fluorescence of the AGE compound 2-(2-furoyl)-4(5)-(2-furanyl)-1 H-imidazole (FFI) was observed at 440 nm after excitation at 375 nm [26]. FN structural modification secondary to advanced glycation was established by assessing bityrosine formation (400 nm upon excitation at 325 nm) and tryptophan quenching (334 nm upon excitation at 275 nm) [27] (Figure 1). Control and AGE-modified FN were evaluated on sodium dodecyl sulfate-polyacrylamide gel electrophoresis (SDS-PAGE) [28] using a 5% stacking gel and a 7.5% separating gel and they exhibited indistinguishable electrophoretic patterns, without any sign of degradation (data not shown). Control and AGE-FN were plated at 4 μg/cm^2^ either onto 12-well tissue culture plates or onto 75 cm^2^ culture flasks for 1 h at room temperature (RT). The plates were gently rinsed three times with PBS before SMCs were allowed to adhere.

### Evaluation of SMC proliferation

An immortalized SMC line (AALTR-16) was kindly provided by Dr. J.K. McDougall (Fred Hutchinson Cancer Research Center, Seattle, WA): human aortic SMCs were infected with a retro viral vector containing the E6/E7 open reading frame of human papilloma virus type 16. The E6 protein binds and promotes the degradation of the wild-type p53 protein and the E7 protein forms an inactivating complex with the product of the retinoblastoma tumor-suppressor gene. These cells did not show any tumorigenic activity in irradiated nude mice [29] and their characterization by immunocytochemical studies and electronic microscopy demonstrated, respectively, expression of alpha -smooth muscle actin and a synthetic phenotype, featured by a prominent endoplasmic reticulum. Lack of oncogenic transformation was evaluated by serum dependent assay (data not shown). The cells were maintained in medium supplemented with 10% FCS, 1 mM l-glutamine, 100 U/mL penicillin and 100 μg/mL streptomycin at 37°C in a humidified atmosphere of 95% air - 5% CO_2_, with medium changes three times a week. Every five days, the cells were harvested with a solution of 0.25% trypsin. To study the effect of AGE-FN on SMC proliferation, cells were seeded at 2 × 10^4^ cells/cm^2^ into 96-well plates coated with either FN or AGE-FN and were cultured in medium supplemented with 10% FCS until sub-confluence. After incubation with serum-free medium for 24-h, cells were incubated for 24 h in serum-free medium (baseline) or in medium containing IGF-I (the concentration of 100 ng/mL was used because a dose–response curve did not show a significant effect of 10, 25 and 50 ng/mL of IGF-I on SMC proliferation [data not shown]). After medium aspiration, fresh medium containing 2 mCi/mL of [methyl-3 H] thymidine (Amersham) was added for additional 4 h. The cells were rinsed twice with ice-cold PBS, twice with ice-cold 5% trichloroacetic acid, and twice with ice-cold 95% ethanol. The cells were lysed in 0.3 mL of 1 N NaOH, neutralized with 0.3 mL of 1 N HCI, and counted in a liquid scintillation counter. The results are expressed as the mean ± SEM of four independent experiments performed in duplicates.

### IGF-I binding assay

SMC were seeded into 12-well plates coated with FN or AGE-FN and were grown in medium supplemented with 10% FCS until sub-confluence. After incubation with serum-free medium for 24-h, the cells were washed with 1 mL of ligand binding buffer and incubated in 0.5 mL of binding buffer containing 25,000 cpm of radiolabeled material (^125^Des (1–3) IGF-I) and unlabeled competitor (Des (1–3) IGF-I) in the following final concentrations: 0, 0.5, 1, 2, 4, 8, 16, 32 and 100 ng/mL as previously described [30]. Total binding was designated as the quantity of labeled ligand bound under these conditions. Nonspecific binding was defined as the radioligand bound in the presence of a 100-fold molar excess of unlabeled ligand. Specific binding was calculated as the difference between total and nonspecific binding.^125^Des (1–3) IGF-I binding kinetics were calculated using a LIGAND computer program [31]. The results are expressed as means ± SEM of three independent experiments performed in duplicates.

### IGF-IR protein content and autophosphorylation

IGF-IR protein levels were determined by immunoblotting membranes containing cell lysates of SMC seeded into 100-mm plates coated with FN or AGE-FN, grown in medium supplemented with 10% FCS until sub-confluence and synchronized by serum starvation for 24-h with antibody 1.2, a monoclonal antibody to the C-terminus of the IGF-IR, as previously described [32]. Three independent experiments were performed. IGF-IR autophosphorylation was analyzed by immunoblotting in cell lysates (75 mg) with an antiphosphotyrosine antibody, as previously described [32]; SMC seed into 100-mm plates coated with FN or AGE-FN were grown in medium supplemented with 10% FCS until sub-confluence and after synchronization for 24-h, cells were incubated either with or without IGF-I (100 ng/mL) for 1 min at 37°C. Three independent experiments were performed.

### IGFBP-4 detection

To study the effect of AGE-modified FN on IGFBP-4 secretion, cells were plated at 3 × 10^4^ cells/cm^2^ into 75 cm^2^ culture flasks coated with FN or AGE-FN, and were grown in medium supplemented with 10% FCS until sub-confluence. After being rinsed twice, they were incubated with serum-free medium supplemented with 1 mM l-glutamine and antibiotics for 24-h. The medium was then replaced with or without addition of PDGF-BB (10 ng/mL) for 24-h. The conditioned medium was collected and concentrated by ultrafiltration in 3 kDa MW cut-off Centripep columns (Amicon Inc., Beverly, MA) and protein was determined by the method described by Bradford [33]. Identification of IGFBP-4 was performed according to the anti-IGFBP-4 antibody manufacturer’s Western Blot (WB) protocol (United Biotech, Inc. Mountain View, CA). Briefly, after gel electrophoresis and transference of proteins, the nitrocellulose membrane was washed twice with water and blocked with phosphate buffered saline (PBS) with 3% non-fat dry milk for 20 min at RT. The membrane was then incubated with a 1:2.000 dilution of antibody anti-human IGFBP-4 (rabbit, polyclonal) in PBS 1% non-fat dry milk at 4°C overnight. This was followed by two washes with water and incubation for 2 h at RT with anti-rabbit immunoglobulin G (peroxidase-linked, Amersham) in PBS 1% non-fat dry milk. The membrane was washed twice with water, once with PBS 0.05% Tween-20 and finally four times with water. A chemiluminescent peroxidase substrate (ECL) was applied for 1 min and the membrane was exposed briefly to autoradiographic film. The densities of the bands were determined by scanning densitometry. Three independent experiments were performed.

### Evaluation of IGF-IR and IGFBP-4 mRNA abundance

To study the effect of AGE-modified FN on IGF-IR and IGFBP-4 mRNA content, total RNA from cells from the previous experiment was prepared using TRIzol® reagent (Invitrogen, Carlsbad, CA) accordingly to standard protocols provided by the manufacturer. RNase protection assay was performed as previously described [34] using the following probes: (I) IGF-IR probe, which is a 379 bp fragment of human IGF-IR cDNA cloned into a pGEM3 vetor [35], (II) IGFBP-4 probe, which is a 505-bp fragment of human IGFBP-4 cDNA cloned into a pBSK + vector [36] and (III) 18S RNA riboprobe, which served as an internal control. Three independent experiments were performed.

### Statistical analysis

Data were analyzed by non-parametric tests. Mann–Whitney test was employed for two unpaired groups and and Kruskal-Wallis test when three or more groups were analyzed. Statistical significance was set to *p* < 0.05. All statistical analyses were performed by JMP Version 5.01 (SAS Institute Inc. Cary, NC, USA).

## Results

### SMC proliferation

**Figure 2:**
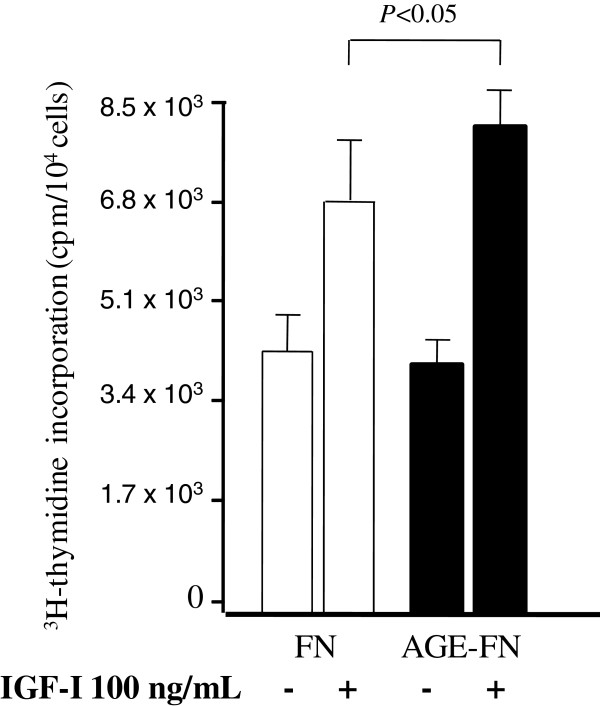
**Effect of the presence (+) or absence (−) of IGF-I (100 ng/mL) on cell proliferation in vascular smooth muscle cells inoculated on fibronectin (FN) and on AGE-modified fibronectin (AGE-FN).** The results are expressed as mean ± SEM of four independent experiments performed in duplicates.

As shown in Figure 2, IGF-I elicited an increment significantly higher (*P* < 0.05) in thymidine incorporation in SMC grown on AGE-FN than in cells grown on FN demonstrating the stimulatory effect of AGE on SMC proliferation in the presence of IGF-I. No differences in thymidine incorporation were observed between SMC coated in FN and AGE-FN in the absence of IGF-I.

### IGF-I binding and IGF-IR autophosphorylation

**Figure 3:**
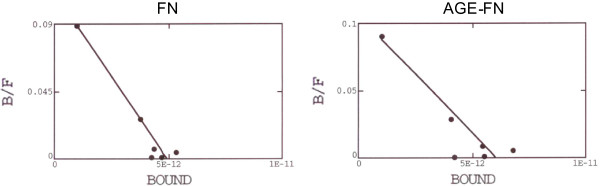
**Scatchard plots of Des (1–3)IGF-I binding sites in vascular smooth muscle cells inoculated on fibronectin (FN) or AGE-modified FN (AGE-FN).** The abscissa shows bound ^125^Des (1–3)IGF-I; the ordinate indicates the ratio of bound (B) Des (1–3)IGF-I to free (F) Des (1–3)IGF-I. A plot representative of three independent experiments is shown.

**Figure 4:**
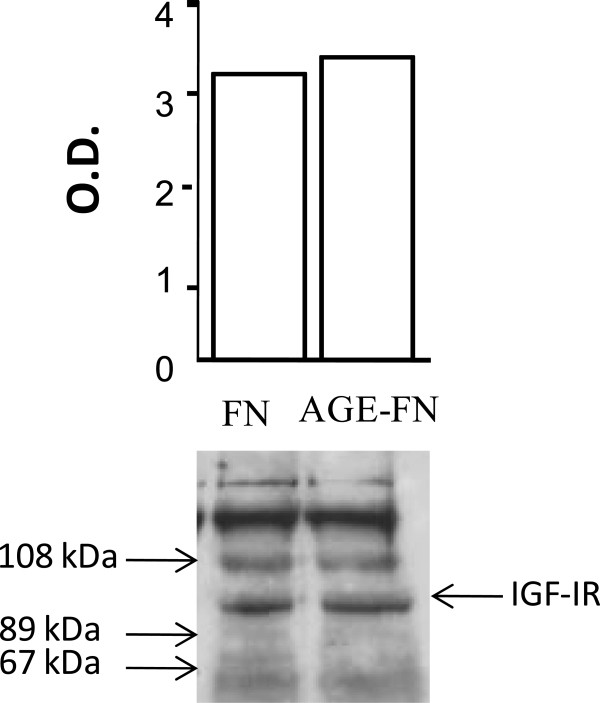
**Western blot analysis of IGF-IR in smooth muscle cells inoculated on fibronectin (FN) and on AGE-modified FN (AGE-FN).** Lysates of cells grown in medium supplemented with 10% FCS until sub-confluence and synchronized by serum starvation for 24-h were analysed using antibody 1.2, a monoclonal antibody to the C-terminus of the IGF-IR. A blot representative of three independent experiments is shown. O.D., optical density.

**Figure 5:**
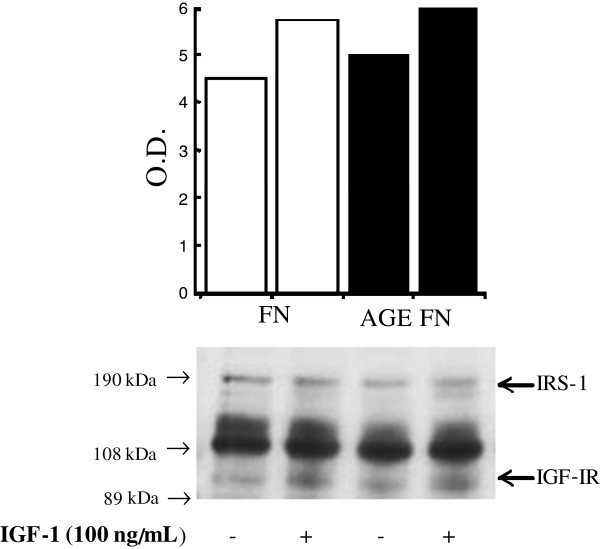
**IGF-I stimulation of tyrosine phosphorylation in smooth muscle cells inoculated on fibronectin (FN) and on AGE-modified fibronectin (AGE-FN).** Lysates of sub-confluent cells exposed (+) or not (−) to IGF-I (100 ng/mL) were analyzed by Western blotting using a antiphosphotyrosine antibody. A blot representative of three independent experiments is shown. O.D., optical density.

In order to investigate if AGE-FN promotes increased SMC proliferation by augmenting IGF-IR number and/or affinity, an IGF-I binding assay was performed with Des(1–3) IGF-I, a truncated form of IGF-I (lacking the N-terminal tripeptide Gly-Pro-Glu) that has much reduced IGFBP affinity compared with natural IGF-I [37]. Thus the use of this analog avoids falsely elevated IGF-IR binding sites, which may occur with any binding of IGF-I radioligand to IGFBPs. IGF-binding curves fitted to one binding site (Figure 3). The receptor numbers per cell were not different between SMC plated on FN and on AGE-FN (1.33 × 10^4^ in both conditions), neither were the relative affinities for IGF-IR (Kd of 6.64 × 10^−11^ and 6.31 × 10^−11^, respectively) (*P* > 0.05). Also, the evaluation of IGF-IR by Western blot did not show any significant difference between the abundance of this protein on SMC grown on FN and AGE-FN (*P* > 0.05) (Figure 4). Since changes in IGF-IR number and affinity could not explain increased cell proliferation stimulated by IGF-I in presence of AGE-FN, autophosphorylation of the IGF-IR was examined by Western blot with an anti-phosphotyrosine antibody. IGF-I stimulated autophosphorylation of SMC seeded on both FN and AGE-FN did not consistently differ between the two experimental conditions (*P* > 0.05) (Figure 5).

### IGFBP-4 content

**Figure 6:**
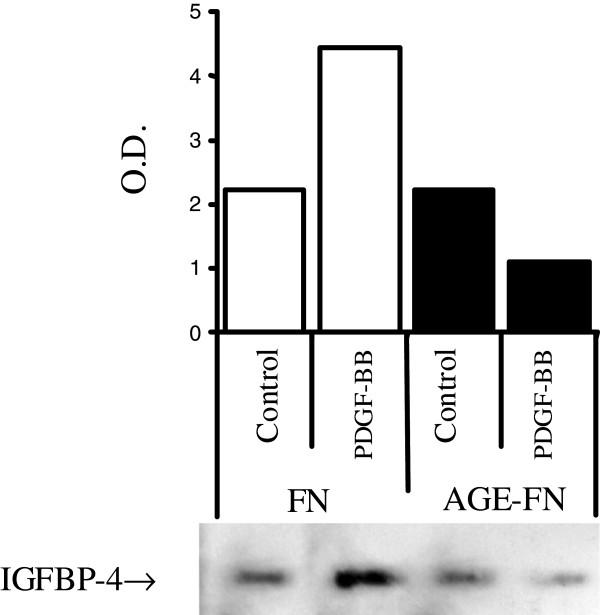
**Western blot analysis of IGFBP-4 in conditioned media of SMC grown on fibronectin (FN) or AGE-modified FN (AGE-FN).** Sub-confluent cells were pre-incubated in serum-free media for 24-h prior to a 24-h exposure to serum-free media (Control) or to 20 ng/mL PDGF-BB (PDGF-BB). A blot representative of three independent experiments is shown. O.D., optical density.

To additionally investigate if a decrease in IGFBP-4, a binding protein that inhibits IGF-I mitogenic effects, was involved in the increased SMC proliferation determined by AGE-FN, a Western blotting employing an antibody anti- human IGFBP-4 was performed. As shown in Figure 6, after 24 h of incubation in serum-free media, the content of IGFBP-4 in the conditioned media of SMC grown on AGE-FN was similar to the observed in the conditioned media of cells seeded on FN (*P* > 0.05), however, treatment with 10 ng/mL of PDGF-BB for 24 h evoked opposite effects on the IGFBP-4 content, augmenting (range from 40–60%) this binding protein in the conditioned media from SMC grown on FN (as observed in cells maintained in plastic substratum [data not shown] and as described in previous studies [22,24]) and decreasing (range from 28–46%) it in the conditioned media of SMC cultivated in AGE-FN (*P* < 0.05). This result suggests that the interaction of SMC with AGE-FN modifies the dynamic of IGFBP-4 secretion induced by PDGF-BB, resulting in elevation of local free IGF-I, which may contribute to SMC proliferation.

### IGF-IR and IGFBP-4 mRNA abundance

**Figure 7:**
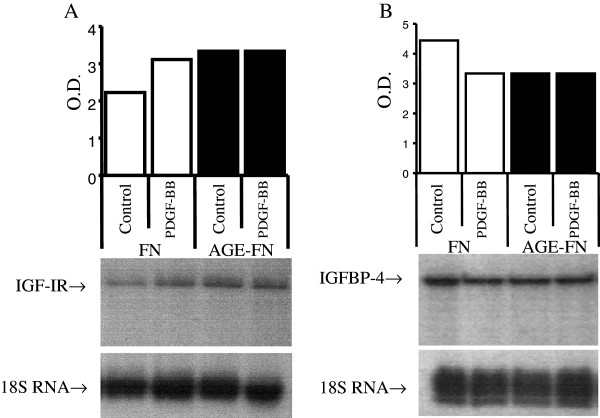
**RNase protection assay analysis of IGF-IR (Panel A) and IGFBP-4 (Panel B) mRNA of SMC grown on fibronectin (FN) or AGE-modified FN (AGE-FN).** Sub-confluent cells were pre-incubated in serum-free media for 24-h prior to a 24-h exposure to serum-free media (Control) or to 20 ng/mL PDGF-BB (PDGF-BB). Twenty micrograms of total RNA were cohybridized with hIGF-IR or hIGFBP-4 and 18S RNA riboprobes. A blot representative of three independent experiments is shown. O.D., optical density.

We next examined the effect of AGE-FN on IGF-IR and IGFBP-4 expression before and after treatment with PDGF-BB, given the known influence of PDGF isoforms on the IGF-I axis in SMC [22,24]. IGF-I expression was not evaluated because preliminary experiments have demonstrated low expression levels of this growth factor in the studied cell line (data not shown). IGF-IR mRNA and IGFBP-4 mRNA abundances were not affected by AGE-FN in comparison to the abundance observed in cells grown on FN substrata after 24 h, and treatment with PDGF-BB had no effect on the content of IGF-IR and IGFBP-4 mRNAs in relation to untreated cells in both substrata (*P* > 0.05) (Figure 7), suggesting that the modulation of IGBBP-4 secretion observed after PDGF-BB treatment is probably due to post-transcriptional events.

## Discussion

There is now a considerable body of evidence supporting an important role for AGEs and its receptors in the diabetic vascular dysfunction [38], such as the suppression of accelerated atherosclerotic lesion development by interruption of AGE-RAGE interaction in diabetic rodents [39]. Our finding of increased IGF-I induced human aortic SMC proliferation in presence of AGE-FN suggests a participation of this growth factor in the atherogenic process related to protein glycation. Given the findings of Chisalita et al. [40], who reported that the expression of IGF-IR is five-fold higher compared to the expression of insulin receptor in human aortic SMC, it is probable that the proliferative effect of IGF-I is being exerted through IGF-IR, although we had not found increased phosphorylation of IGF-IR.

Previous studies have already demonstrated a stimulatory effect of AGE on the growth of rat [41], rabbit [42], porcine [43] and human [44] vascular SMC. Seki et al. [45], who also reported a proliferative effect of AGE on rat SMC, did not detect any change in the phosphorylation of PDGF receptor. In common with the present study, SMC were chronically maintained in AGE and were acutely stimulated with the growth factor in the phosphorylation experiment. Thus, these two studies suggest that chronic exposure of SMC to AGE did not modify the phosphorylation status of PDGF and IGF receptors, although it positively affected cell proliferation. On the other hand, activation of p42 mitogen-activated protein kinase (MAPK) [42] and of p21^ras^/MAP kinase pathway [46] were described in SMC acutely exposed to AGE, demonstrating the direct activation of these transduction pathways by AGE-RAGE interactions.

Human vascular SMCs synthesize and secrete IGFBP-2, -4, -5 and −6. Their expression is regulated by many factors, including PDGF, and they are important determinants of SMC responses to IGF-I [47] inhibiting (IGFBP-4) or potentiating (IGFBP-5) its mitogenic effect [48]. We evaluated IGFBP-4, the most abundant binding protein secreted by human SMC and found that AGE-FN modifies its modulation by PDGF-BB, decreasing IGFBP-4 production in comparison to FN substratum, in which PDGF-BB elicits an increased secretion. Since IGFBP-4 is known to inhibit IGF-I action, the reduction of IGFBP-4 secretion induced by PDGF-BB could result in augmented availability of IGF-I. It is well known that the functions of IGFBPs on vascular SMC are regulated by post-translational processing such as proteolysis [47]. In the case of IGFBP-4, the metalloproteinase pregnancy-associated plasma protein A (PAPP-A), expressed in vascular SMC [49], is recognized as the main protease. It remains to be elucidated if, in the presence of AGE-FN, PDGF-BB could induce PAPP-A expression, increasing IGFBP-4 proteolysis and eventually decreasing the abundance of this binding protein. This is an interesting possibility in light of previous works demonstrating that PAPP-A expression is stimulated by TNF-α in human coronary SMC [50] and in human fibroblasts [51] and that, in the latter cell type, NFκB was identified as the primary mediator of TNF-α stimulated PAPP-A gene expression [52]. Both TNF-α [10] and NFκB [13,45] are stimulated after cellular exposure to AGE-modified proteins.

Since we did not perform experiments evaluating autophosphorylation of PDGF-BB receptor, we cannot rule out that a decrease in PDGF-BB receptor signaling is taking place in presence of AGE-FN, which could partially explain an absence of PDGF-BB effect increasing IGFBP-4 in the conditioned media. A previous study of Cantero et al. found that dicarbonyl compounds, highly reactive compounds that react with proteins to form AGEs, modified PDGF-induced PDGFRβ-phosphorylation by inhibiting its intrinsic tyrosine kinase activity [53].

## Conclusions

This study suggests that AGE-modified FN modulates SMC’s response to IGF-I and PDGF-BB, both present in the vascular wall, potentially contributing to the pathogenesis of diabetes associated atherosclerosis by increasing SMC susceptibility to IGF-I mitogenic effects.

## Abbreviations

AGE: Advanced glycation end products; AGE-FN: Advanced glycation end product-modified fibronectin; IGF: Insulin-like growth factor; IGFBP: Insulin-like growth factor binding protein; FN: Fibronectin; PAPP-A: Pregnancy-associated plasma protein A; PDGF: Platelet-derived growth factor; SMC: Smooth muscle cells.

## Competing interests

The authors declare that there is no competing interests that would prejudice the impartiality of this scientific work.

## Authors’ contributions

MLCG performed the experiments and prepared the manuscript, MRAZ contributed to experiments, DLR provided reagents and advice and reviewed the paper, DGN designed the study, obtained funding, analyzed the data and reviewed the paper. All authors read and approved the final manuscript.
